# Engineering the pathway in *Escherichia coli* for the synthesis of medium-chain-length polyhydroxyalkanoates consisting of both even- and odd-chain monomers

**DOI:** 10.1186/s12934-019-1186-x

**Published:** 2019-08-13

**Authors:** Qianqian Zhuang, Qingsheng Qi

**Affiliations:** 1 State Key Laboratory of Biobased Material and Green Papermaking, Qilu University of Technology (Shandong Academy of Sciences), Jinan, 250353 People’s Republic of China; 2 Shandong Provincial Key Laboratory of Microbial Engineering, School of Bioengineering, Qilu University of Technology (Shandong Academy of Sciences), Jinan, 250353 People’s Republic of China; 3 State Key Laboratory of Microbial Technology, Shandong University, Qingdao, 266237 People’s Republic of China

**Keywords:** *Escherichia coli*, Polyhydroxyalkanoates, Odd-chain monomers, Reversed fatty acid β-oxidation cycle, Metabolic engineering, Synthetic biology

## Abstract

**Background:**

Medium-chain-length polyhydroxyalkanoates (mcl-PHAs) containing various chain length monomers from C6 to C14 have more applications besides sustainable and environmental-friendly biomaterials owing to their superior physical and mechanical properties. We engineered a reversed fatty acid β-oxidation pathway in *Escherichia coli* that can synthesize mcl-PHA directly from glucose and achieved high yield. However, there were only even-chain monomers in the biosynthetic polymers. The need for mcl-PHA harboring both even- and odd-chain monomers with better and wider utility impels us to develop the biosynthetic routes for the production of the novel and unnatural mcl-PHA through rewiring the basic metabolism.

**Results:**

In the present study, a propionate assimilation and metabolic route was integrated into the reversed fatty acid β-oxidation in order to produce mcl-PHA consisting of both even- and odd-numbered monomers. The content of odd-numbered monomers in mcl-PHA was improved with the increased propionate addition. After further deletion of pyruvate oxidase (PoxB) and pyruvate formate-lyase (PflB), the metabolically engineered chassis *E. coli* LZ08 harboring pQQ05 and pZQ06 (overexpression of *prpP* and *prpE* genes from *R*alstonia *eutropha* H16) innovatively accumulated 6.23 wt% mcl-PHA containing odd-chain monomers ranging from 7 to 13 carbon atoms about 20.03 mol%.

**Conclusions:**

This is the first successful report on production of mcl-PHA harboring both even- and odd-chain monomers (C6–C14) synthesized from glucose and propionate in recombinant *E. coli*. This present study achieved the highest yield of de novo production of mcl-PHA containing odd-numbered monomers in *E. coli* at shake-flask fermentation level. Continued engineering of host strains and pathway enzymes will ultimately lead to more economical production of odd-chain monomers based on market demand. The synthetic pathway can provide a promising platform for production of other value-added chemicals and biomaterials that use acetyl-CoA and propionyl-CoA as versatile precursors and can be extended to other microorganisms as intelligent cell factories.

**Electronic supplementary material:**

The online version of this article (10.1186/s12934-019-1186-x) contains supplementary material, which is available to authorized users.

## Background

The continuous consumption of resources such as petroleum and fossil fuels along with the increasing environmental pollution caused by petrochemical plastics have generated significant interests in developing and synthesizing bio-based materials. Polyhydroxyalkanoates (PHAs), as a class of environmental-friendly biomaterials, are accumulated by a variety of microbes from renewable carbon resources such as sugars [1, 2]. They have garnered great attention because of their unparalleled properties similar to elastomers and thermoplastics as potential alternatives for petroleum-based polymers [3, 4].

According to the different chain length monomer composition, PHAs can be divided into three main types: short-chain-length PHAs (scl-PHAs) which contain 3–5 carbon atoms, medium-chain-length PHAs (mcl-PHAs) which contain 6–14 carbon atoms, and scl-mcl PHAs which contain 3–14 carbons in length [5]. The composition of copolymers determines the physical and mechanical material properties of the bioplastics. Generally, mcl-PHAs are synthesized via fatty acid de novo biosynthesis pathway or β-oxidation pathway from *Pseudomonads* in nature [6]. They are semicrystalline and thermoplastic elastomers which are suitable for the materials in biomedical application [7]. The traditional mcl-PHAs with only even-chain monomers have shown to own a desirable set of physical properties, and incorporating the fractions of odd-numbered monomers may lend the plastics more strength and flexibility so as to endow the polyesters novel and favorable properties and utilities. The *Pseudomonas putida* KT2442 mutant, KTOY06, accumulated a homopolymer of poly-3-hydroxyheptanoate (P3HHp) up to 71 wt% of its cell dry weight (CDW) when heptanoate was added as a single carbon source [8]. In another case, 3-hydroxynonanoate (3HN) monomer (30–80 mol%) was the major constituent of polyhydroxyalkanoates accumulated from odd-numbered fatty acids by microorganisms [9]. Lately, feeding of odd carboxylic acids ranging from valeric acid to pentadecanoic acid resulted in the odd carbon number monomer fractions such as 3HHp, 3HN and 3-hydroxyundecanoate (3HUD) and a small amount (10 mol% or less) of even carbon number monomer fractions was also detected in *P. putida* Bet001 [10]. Researchers also reported that in N-limited shake flasks using nonanoic acid, *P. citronellolis* DSM 50332 produced 32% of its dry biomass as mcl-PHA containing 78% 3HN with 22% 3HHp [11]. Therefore, propionate or odd-chain fatty-acid-rich feedstocks have been exogenously supplemented in the culture medium for their direct conversion to propionyl-CoA as the aforementioned studies. However, the high costs and toxicity to microbial cells associated with these fatty acids will limit their practical applications. Besides, the monomer types of mcl-PHA synthesized in the above research were not diversified. In view of this, it is a pressing demand to exploit an efficient metabolic pathway that leads to the formation of corresponding odd-chain (R)-3-hydroxyacyl-CoA as precursors for the acyl-chain elongation to biosynthesize mcl-PHA containing various odd-numbered monomers via adding the inexpensive carbon source-glucose.

For the past few years, rational strategies for metabolic pathway engineering and synthetic biology were exploited to balance the enzyme expression, eliminate the pathway regulatory bottleneck, and facilitate the production of targeted metabolites [12–16], such as PHA production [17–20]. The engineered reversal of the fatty acid β-oxidation cycle provides a promising platform that can support the generation of various advanced products at high yields from renewable feedstocks recently with the development of systems metabolic engineering and synthetic biology [21–23]. Furthermore, there has been no report on the accumulation of odd-chain acyl-CoA for mcl-PHA production using glucose and propionate in *E. coli* cell factory by far. For this reason, the functional fatty acid β-oxidation reversal was mediated through supplying two-carbon extending acyl-CoA molecules from unrelated and cheap carbon source as biogenic precursors to synthesize different odd-numbered (R)-3-hydroxyacyl-CoA instead of adding only related carbon sources-fatty acids. To synthesize mcl-PHA that contained odd-chain monomers from the reversed fatty acid β-oxidation cycle, the starting precursor propionyl-CoA must be provided. In the previous study, after overexpressing the *prpP* gene in *E. coli*, the increasing pool of intracellular propionate facilitated the content of propionyl-CoA and increased the cell biomass [24]. For the production of PHBV, Yang et al. employed the *prpE* gene from *Ralstonia eutropha* H16 to synthesize the propionyl-CoA and elevated the 3HV monomer fraction [25]. Regarding Pct_Re_, it can catalyze the transfer of CoA from acetyl-CoA to propionate [26]. At the same time, it is worth mentioning that acetate overflow is the major drawback for production of acetyl-CoA-derived chemicals. Approaches for overcoming acetate overflow may be beneficial for biomass accumulation and the production of acetyl-CoA-derived products; for instance, PHA [27]. This research aimed to construct the metabolic pathway for PHA production by integrating two parallel modules leading to the production of the even-chain monomers and the odd-chain monomers. The results demonstrated that the amount of odd-numbered monomers accumulated in the recombinant *E. coli* depended on the combination of propionate supplementation and propionyl-CoA supply. This is the first case revealing that engineered *E. coli* can produce novel and unnatural mcl-PHA consisting of the highest amount of odd-chain ranging from C7 to C13 motieties from glucose with addition of propionate.

## Results and discussion

### Construction and integration of individual module to enable direct microbial synthesis of even- and odd-chain mcl-PHA

**Fig. 1:**
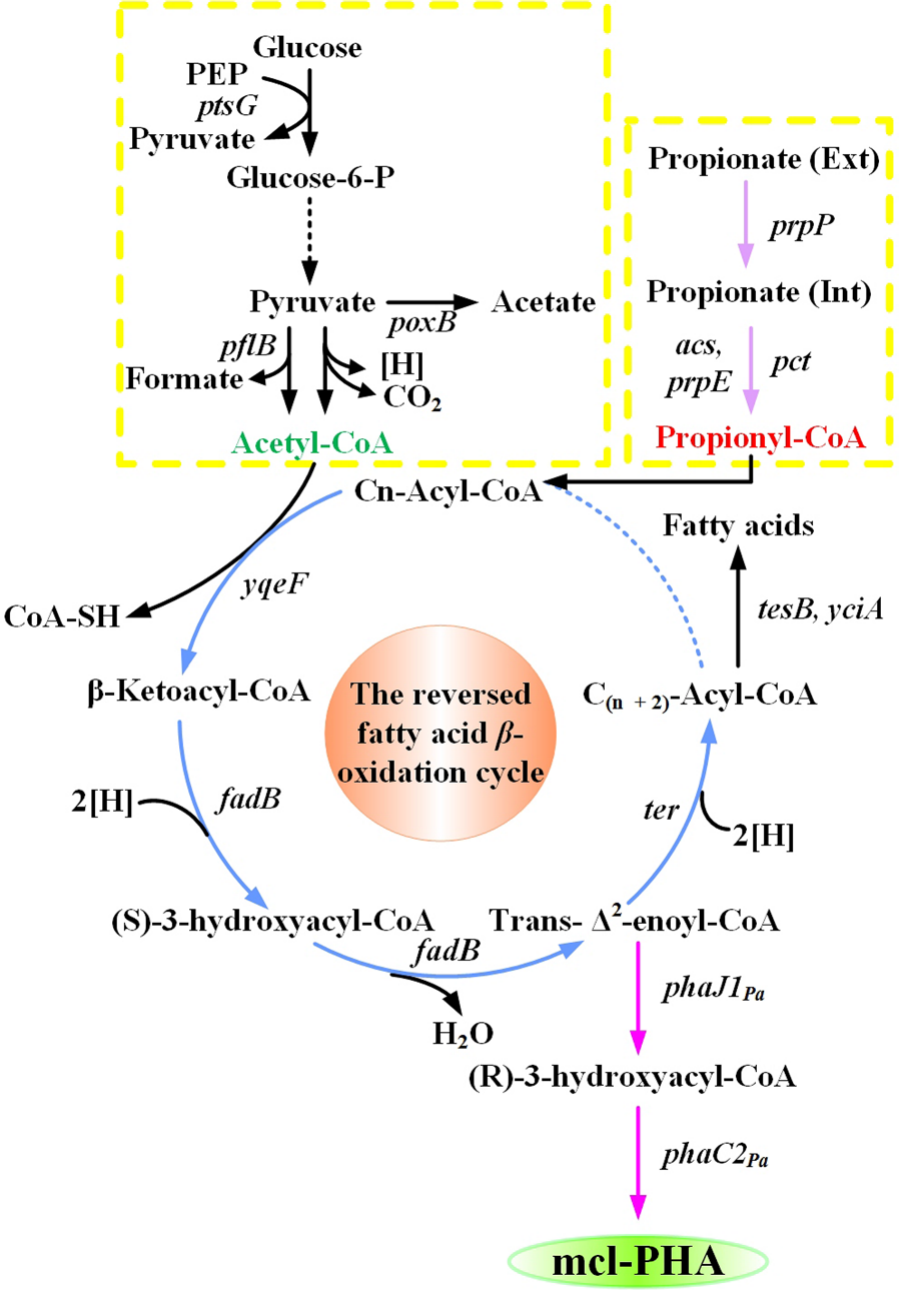
Schematic pathway for mcl-PHA biosynthesis consisting of odd-chain and even-chain monomers. Genes overexpressed or deleted and metabolic intermediates in the recombinant *E. coli* are marked. After every cycle, two carbons were added to the initial acyl-CoA thioester (indicated as Cn + 2) was generated. Dashed lines indicate multiple steps. Dashed lines without arrowheads connect acyl-CoA intermediates of different chain length. Genes in bold were expressed to obtain mcl-PHAs from the reversed β-oxidation cycle. Marked genes: *ptsG*, glucose-specific PTS permease, IIBC component; *poxB*: pyruvate oxidase; *pflB*: pyruvate formate-lyase; *yqeF*, thiolase; *fadB*, hydroxyacyl-CoA dehydrogenase/enoyl-CoA hydratase; *ter*, trans-2-enoyl-CoA reductase from *T. denticola*; *phaJ1*_*Pa*_, (R)-specific enoyl-CoA hydratase from *P. aeruginosa* PAO1; *phaC2*_*Pa*_, polyhydroxyalkanoic acid synthase from *P. aeruginosa* PAO1; *tesB/yciA*, thioesterase; *prpP*, propionate permease; *acs*, acetyl-CoA synthase; *prpE*, propionyl-CoA synthase; *pct*, propionate-CoA transferase. The left yellow dotted box indicates the even-chain precursor (acetyl-CoA) supply module. Purple arrows indicate the odd-chain precursor (propionyl-CoA) supply module in the right yellow dotted box. Blue arrows indicate the reversed β-oxidation cycle module

In the previous study, we constructed an efficient even-chain mcl-PHA biosynthetic pathway in recombinant *E. coli* via the reversed fatty acid β-oxidation cycle [23]. In order to supply odd-chain monomer precursors, an intracellular de novo biosynthetic module was established. For this, *prpP*, *acs*, *prpE*, and *pct* genes were overexpressed and then we constructed a series of plasmids, namely pZQ01 (pBBR1MCS2-prpP), pZQ02 (pBBR1MCS2-acs), pZQ03 (pBBR1MCS2-prpE) and pZQ04 (pBBR1MCS2-pct) as four kinds of odd-chain precursor supply module. After this, the entire pathway was built and divided into three modules: even-chain precursor supply, odd-chain precursor supply, and the reversed β-oxidation biosynthetic cycle (Fig. 1). Glucose leads to the formation of acetyl-CoA through glycolysis for the priming molecule production whereas in the engineered pathway of odd-chain precursor supply, propionate uptake can be promoted by propionate permease (PrpP) according to the previous results [24]. Meanwhile, propionate is also directly activated by propionyl-CoA synthetase (PrpE/Acs) or propionate CoA-transferase (Pct) to incorporate a 3-hydroxyvaleryl-CoA unit into the extended polymer chain by the PHA synthase encoded by *phaC2*_*Pa*_ from *Pseudomonas aeruginosa* PAO1. In light of this reason, the *prpE* gene encoding propionyl-CoA synthase from *R. eutropha* H16 was used to attach CoA group to propionic acid. Overexpression of *prpP* increased the substrate of PrpE to form propionyl-CoA. Additionally, Liu et al. found that deletion of *acs* in *E. coli* resulted in blockage of propionate utilization, so that Acs is essential for propionate utilization in *E. coli* and responsible for transforming propionate to propionyl-CoA [28]. The propionate CoA-transferase from *R. eutropha* H16 (Pct_Re_) preferably uses propionate as CoA acceptor [29].

**Fig. 2:**
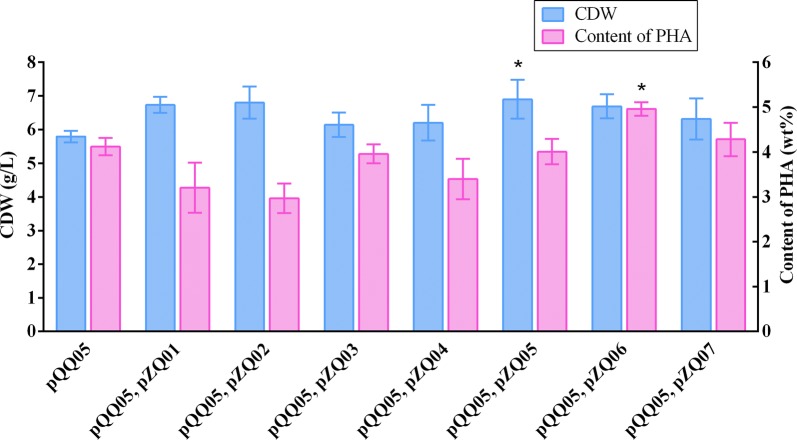
The cell dry weight and content of mcl-PHA in recombinant *E. coli* LZ05 harboring various combination of plasmids. The experiments were performed in triplicate, and error bars indicate standard deviation (SD). The * denotes *P* < 0.05 compared with the control strain LZ05/pQQ05

**Fig. 3:**
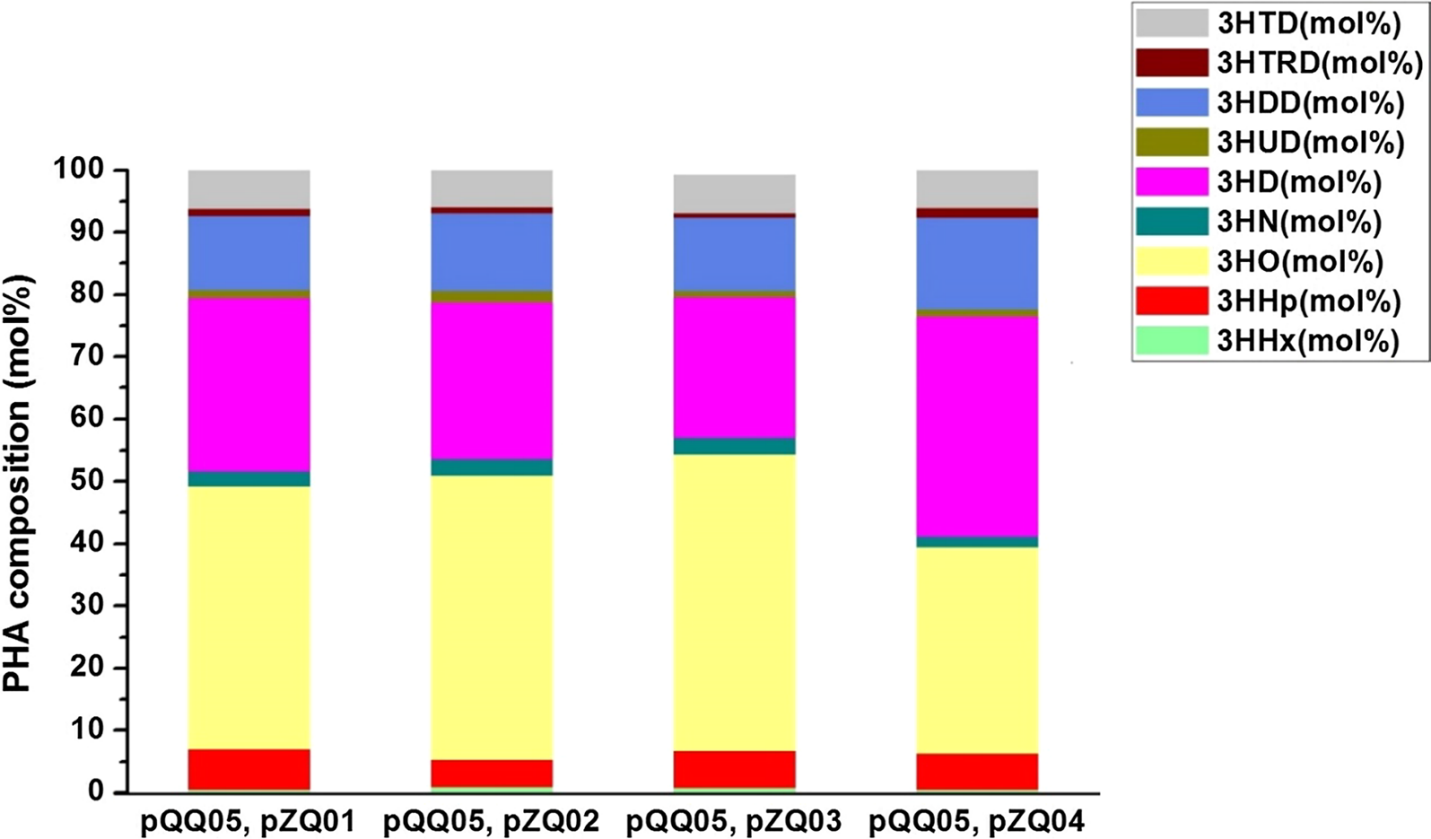
Composition of mcl-PHA in recombinant *E. coli* LZ05 harboring various combination of plasmids. All data were the average of three independent studies

After integration of the aforementioned three modules, we utilized them together to produce the even- and odd-chain mcl-PHA polyesters from glucose with supplement of extracellular propionate. To assess the effect of genes *prpP*, *acs*, *prpE* and *pct* on the odd-chain fractions in mcl-PHA production, we co-transformed the plasmids pQQ05 and pZQ01, pQQ05 and pZQ02, pQQ05 and pZQ03, pQQ05 and pZQ04, respectively, into the engineered *E. coli* strain LZ05 which yielded the highest content of even-chain mcl-PHA and set LZ05 containing pQQ05 (LZ05/pQQ05), previously constructed, as the control strain [23]. After the shake flask study at 30 °C and 250 rpm by co-feeding 30 g/L glucose and 1.5 g/L propionate, the control strain LZ05/pQQ05 was able to synthesize about 4.12 wt% mcl-PHA only with even-chain monomers (Fig. 2). However, the recombinant strain LZ05 harboring the above combination of plasmids were all found to be able to accumulate even- and odd-numbered mcl-PHA with carbon chain length from C6 to C14. The results suggested that we successfully established the distinct pathway for producing targeted mcl-PHA in *E. coli*. Among these recombinants, the strain LZ05 harboring plasmids pQQ05 and pZQ02 accumulated the mcl-PHA approximately 2.97 wt% containing the highest amount of even-chain monomers about 90.17 mol%. Moreover, LZ05 (pQQ05, pZQ02) also produced the higher molar content of 3-hydroxyhexanoate (3HHx) fraction than that of other recombinants. Furthermore, the engineered strain LZ05 harboring plasmids pQQ05 and pZQ01 produced mcl-PHA approximately 3.21% of the CDW with the highest amount of odd-numbered mcl-PHA monomers up to 11.24 mol%, among which the 3HHp fraction in mcl-PHA was maintained at about 6.46 mol%. It indicated that a relatively higher metabolic flux was shunted towards propionyl-CoA synthesis when overexpressing *prpP* in the cells (Figs. 2, 3). Nevertheless, the engineered strain LZ05 harboring plasmids pQQ05 and pZQ03 resulted in about 3.96% mcl-PHA of the CDW, which was the highest PHA content compared with the other three combination of plasmids. The molar ratios of 3HHx, 3HHp, 3-hydroxyoctanoate (3HO), 3HN, 3-hydroxydecanoate (3HD), 3HUD, 3- hydroxydodecanoate (3HDD), 3-hydroxytridecanoate (3HTRD) and 3-hydroxytetradecanoate (3HTD) were approximately 0.76, 5.91, 48.60, 2.82, 22.49, 0.98, 11.74, 0.69 and 6.01, respectively. In total, it had the odd-chain monomers with a molar content of 10.40% (Figs. 2, 3). Herein, it can be concluded that *prpP* increased odd-numbered fraction formation rate and stimulate the cells to use more propionate for odd-chain fraction yield, although it may not accumulate the highest amount of mcl-PHA polyesters. Regarding the cell growth phenomena, LZ05 harboring plasmids pQQ05 and pZQ02 had the best performance and reached the cell dry weight of 6.80 g/L, while the CDW of the control strain LZ05/pQQ05 could only reach about 5.79 g/L. As for the composition of mcl-PHA, except the strain LZ05 (pQQ05, pZQ04) harboring mainly 3HD fraction, the other three constructed strains are all composed primarily of 3HO monomer (Figs. 2, 3). These results demonstrated that genes *prpP*, *acs*, *prpE* and *pct* had diverse impacts on the mcl-PHA production. Overexpression of *prpP* gene indeed enhanced the transport efficiency of propionate and offered more precursors for propionyl-CoA. Although overexpressing *prpE* gene accumulated the odd-chain monomers and obtained the relatively higher 3HHp and 3HN content, the incorporated odd-chain fraction was still rather low. In addition, when we overexpressed *pct* in pZQ04, 10.36 mol% of odd-chain monomers were detected. This is most likely because Pct harnessed acetyl-CoA and propionate as substrates to form propionyl-CoA, resulting in precursors for initiation of the odd-chain monomer biosynthesis. According to these experimental results, the strains still did not have sufficient molecules for initiation the formation of odd-chain monomers.

### Improvement of odd-chain monomer biosynthesis by simultaneous overexpression of double genes in the metabolic pathway

**Fig. 4:**
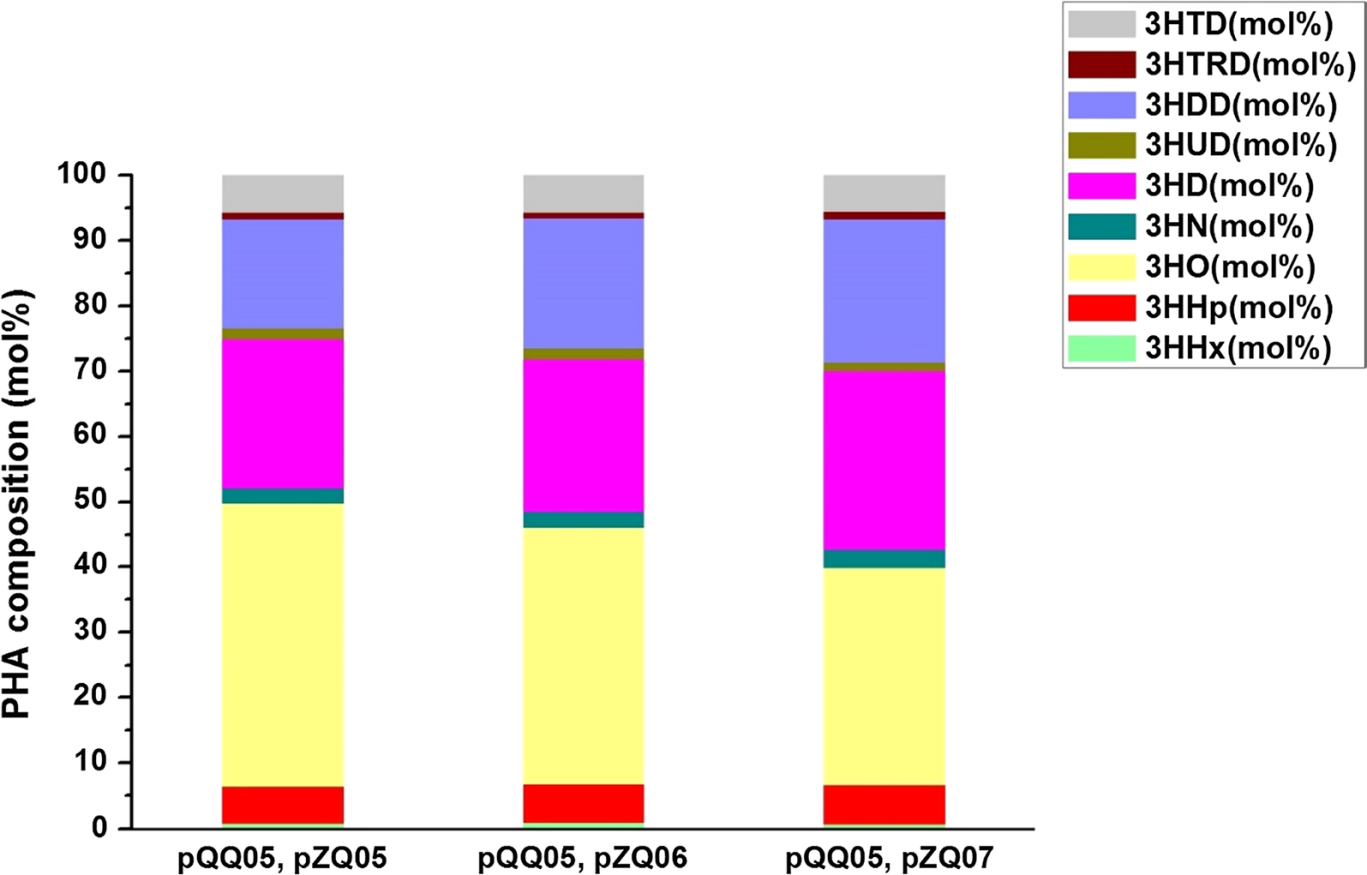
Composition of mcl-PHA in recombinant *E. coli* LZ05 harboring novel combination of plasmids. All data were the average of three independent studies

To increase the mcl-PHA production and possess more odd-numbered monomers in recombinant *E. coli*, we rebuilt the biosynthetic pathway by construction of the plasmids pZQ05, pZQ06 and pZQ07. In the above plasmids, *prpP* and *acs*, *prpP* and *prpE*, and *prpP* and *pct* were simultaneously overexpressed separately, due to the highest gain of odd-numbered monomers when overexpression of *prpP*. Then, they were all co-transformed with pQQ05 into the strain LZ05 to form LZ05 (pQQ05, pZQ05), LZ05 (pQQ05, pZQ06), and LZ05 (pQQ05, pZQ07). The shake flask study was conducted at 30 °C and 250 rpm with addition of 30 g/L glucose and 1.5 g/L propionate. Cultivation of these strains and the control strain LZ05/pQQ05 showed that they exhibited diverse growth phenomena and different accumulation of mcl-PHA with odd-numbered monomers. The CDW of LZ05 harboring double plasmids pQQ05 and pZQ05 reached approximately up to 6.90 g/L and the cellular dry weight of LZ05 harboring double plasmids pQQ05 and pZQ06 (or pZQ07) reached about 6.69 g/L (or 6.31 g/L). Nevertheless, the CDW of LZ05/pQQ05 could reach 6.62 g/L when we simply added 30 g/L glucose for the shake flask study under the same conditions with this research in our previous work [23]. In order to verify the effect of the single gene or three gene combination (*prpP* and *acs*, *prpP* and *prpE*, and *prpP* and *pct*) to the cell growth, statistical analyses were carried out. The results of significance tests denoted the significant difference in the CDW between LZ05 (pQQ05, pZQ06) and the control strain LZ05/pQQ05 (*P* < 0.05, Fig. 2). Moreover, it also demonstrated that co-overexpression of *prpP* and *acs* restored the cell growth compared with that of the strain LZ05/pQQ05 without propionate addition (6.62 g/L). As a result, overexpressing *prpP* and *acs* not only offset the toxicity of propionate to cells, but also balanced the carbon flux between cell growth and PHA synthesis. With regard to the content of mcl-PHA, they had different reflection in different strains. LZ05 (pQQ05, pZQ06) accumulated 4.96 wt% mcl-PHA polymers which is the highest among the aforementioned three strains and resulted in 1.2 fold (*P* < 0.05) increase in mcl-PHA production compared to the control strain. Similarly, the content of mcl-PHA was also enhanced by 1.6-fold (*P* < 0.001), 1.7-fold (*P* < 0.0001), 1.3 fold (*P* < 0.05), and 1.5-fold (*P* < 0.001) compared with LZ05 (pQQ05, pZQ01), LZ05 (pQQ05, pZQ02), LZ05 (pQQ05, pZQ03), and LZ05 (pQQ05, pZQ04), respectively. Nevertheless, the molecular content of odd-numbered fractions in LZ05 (pQQ05, pZQ06) of about 10.98 mol% was lower than LZ05 (pQQ05, pZQ01). Reasons for this phenomenon could be attributed to two points: the first one is the proper expression level of *prpE* is vital for its high activity to obtain high content of odd-numbered fractions. It has been reported that the expression of *prpE* from *Salmonella enterica* at a relatively high level led to decrease on 3HV content in the PHBV [30]. The second one is whether the substrate of PrpE-propionate addition is sufficient or not. It is important to engineer host strains to provide enough precursors of specific HA-CoAs with PHA synthase for efficient production of mcl-PHA containing corresponding monomer constituents. To verify this, we attempted to supplement different concentrations of propionate in the subsequent research. Additionally, increasing total amount of mcl-PHA heterogeneous polymers was detected in the two strains LZ05 harboring pQQ05 and pZQ05, and LZ05 containing pQQ05 and pZQ07 which accounted for 4.01 wt% and 4.28 wt%, respectively. Compared with LZ05 (pQQ05, pZQ01) and LZ05 (pQQ05, pZQ04), 3HN and 3HUD fractions were augmented up to 2.77 mol% and 1.27 mol% after both overexpression of *prpP* and *pct* in the strain LZ05 (pQQ05, pZQ07) (Figs. 2, 4). This study also demonstrated that genetically modulating propionyl-CoA metabolism can be potentially applied to tailor the monomer fraction of the PHA copolymer and have preferable properties for various applications.

### Effect of propionate concentration on odd-chain monomer production

**Fig. 5:**
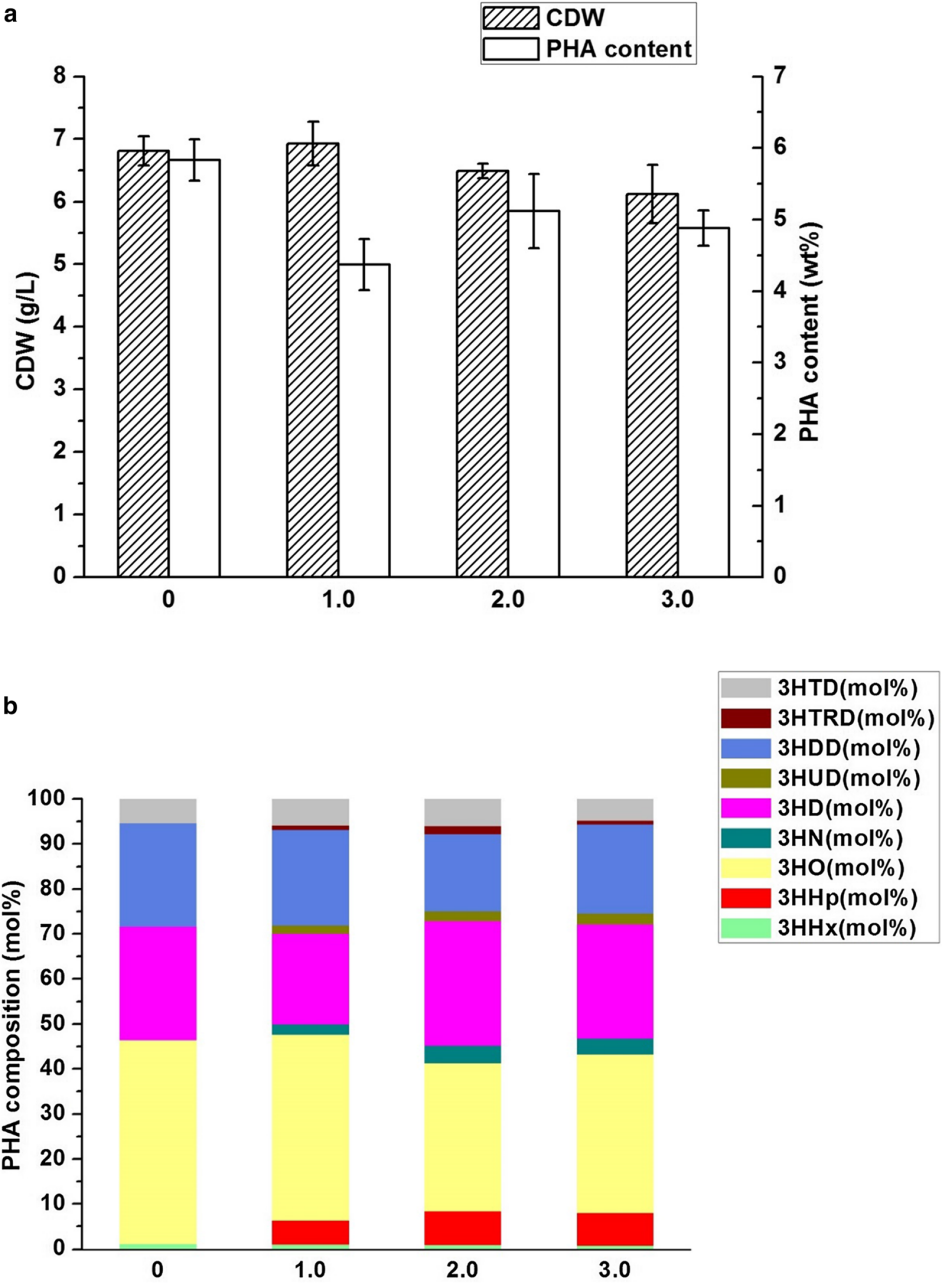
The effects of propionate at different concentrations on the mcl-PHA production of *E. coli* LZ05 harboring pQQ05 and pZQ06. **a** The CDW and PHA content of the engineered *E. coli*; **b** composition of mcl-PHA in the engineered *E. coli*. Fermentations were conducted in shake flasks at 30 °C and 250 rpm with 30 g/L glucose. The experiments were performed in triplicate, and error bars indicate standard deviation (SD)

In order to enhance the content of the odd-numbered fractions in the strain LZ05 harboring pQQ05 and pZQ06, propionate of various concentrations was successively added to the medium to examine the effects. The results showed that the addition of propionate was essential for the production of odd-numbered monomers in mcl-PHA copolymers, and also able to substantially promote the proportion of odd-numbered monomers in mcl-PHA copolymers with the most suitable propionate concentration. On one hand, when the concentration of propionate in the medium increased from 0 to 2.0 g/L, the proportion of odd-numbered monomers in the mcl-PHA increased from 0 to 15.30 mol%, on the other hand, the proportion of even-numbered monomers in the mcl-PHA decreased from 100 to 84.70 mol%. The concentration of mcl-PHA and CDW were approximately 5.12 wt% and 6.49 g/L, respectively, when the concentration of propionate was 2.0 g/L. However, when the concentration of propionate in the medium increased to 3.0 g/L, the proportion of odd-numbered monomers in the mcl-PHA decreased to 14.15 mol%. Meanwhile, the concentration of mcl-PHA and biomass decreased to 4.88 wt% and 6.12 g/L (Fig. 5a, b). This suggested that the high concentration of propionate inhibited cell growth, leading to a reduction of odd-numbered monomer content and total mcl-PHA production. Therefore, the optimal concentration of propionate in the medium was 2.0 g/L. With this concentration, the monomer content of LZ05 (pQQ05, pZQ06), 3HHp, 3HN, 3HUD and 3HTRD fractions were all enhanced to 7.42 mol%, 3.88 mol%, 2.20 mol% and 1.80 mol%, respectively. As a whole, not only was the engineered *E. coli* LZ05 (pQQ05, pZQ06) capable of obtaining the highest odd-numbered fraction content, but the total mcl-PHA content in the cell dry weight was also promoted.

### Improvement of mcl-PHA accumulation by reinforcing acetyl-CoA supply

**Fig. 6:**
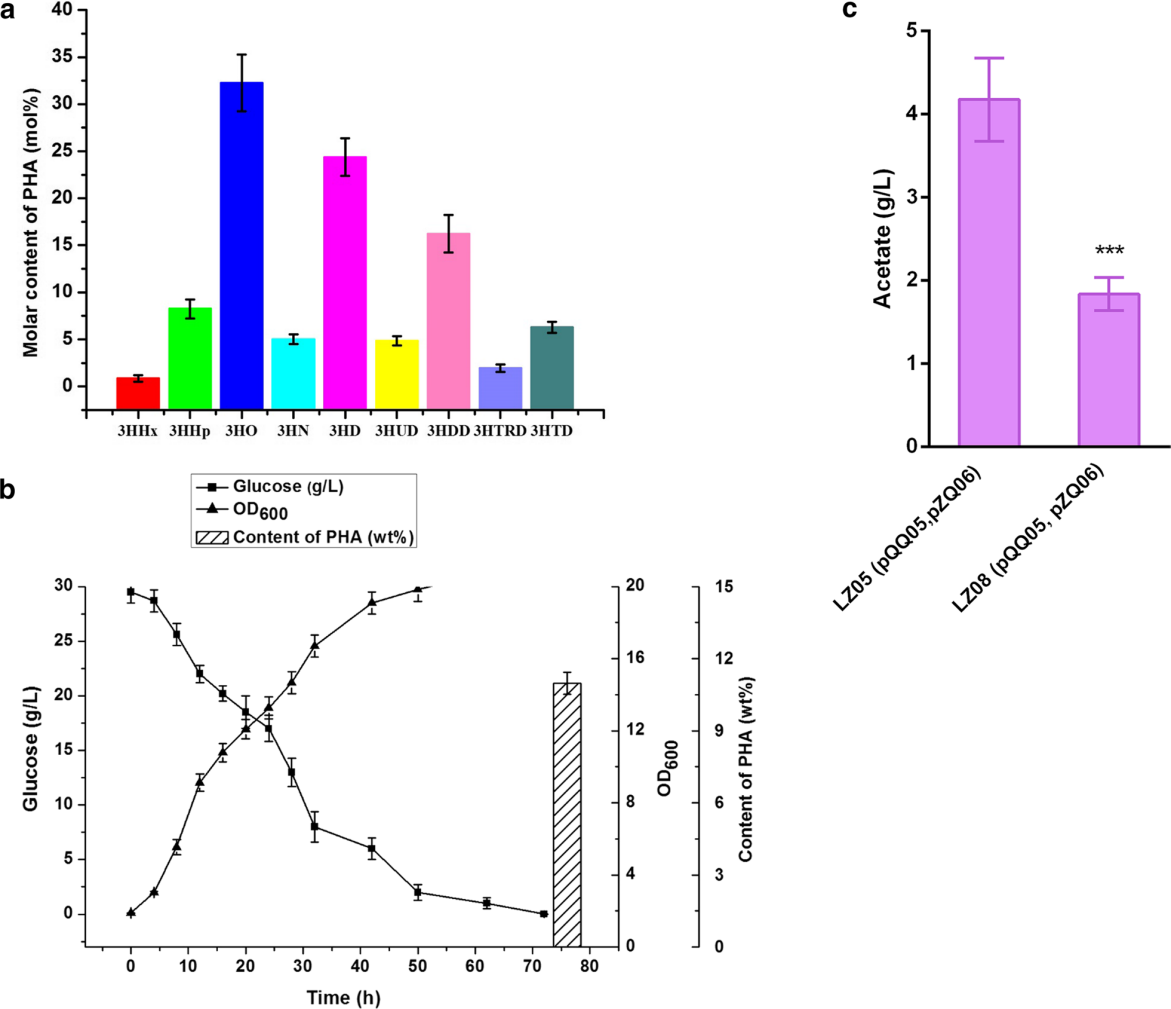
Fermentation profiles of the *E. coli* LZ08 harboring plasmids pQQ05 and pZQ06 and mcl-PHA production. **a** Monomer composition and molar content of accumulated PHA. The percentage ratios of PHA produced are also shown. **b** Strain growth and glucose consumption were displayed in curve graph, while total content of PHA of the recombinant strain were determined at the end of fermentation and were indicated using histogram. **c** Acetate secretion of LZ05 (pQQ05, pZQ06) and LZ08 (pQQ05, pZQ06). Fermentations were conducted in shake flasks at 30 °C and 250 rpm with 30 g/L glucose and 2.0 g/L propionate. The experiments were performed in triplicate, and error bars indicate standard deviation (SD). Statistical analysis on the figure shows comparison of acetate secretion between LZ05 (pQQ05, pZQ06) and LZ08 (pQQ05, pZQ06). The *** indicate *P* < 0.001

Acetyl-CoA is a key molecule in microbial central carbon metabolism and is the direct initial molecule taking part in mcl-PHA biosynthetic pathway, so enhancement of acetyl-CoA supply shall promote the mcl-PHA formation. When glucose is excess in the medium, *E. coli* cells do not fully activate TCA cycle, and thus generate several by-products, such as acetate [31]. The acetate secretion not only caused low pH and was toxic to cell growth, but also decreased acetyl-CoA pool and the formation of target products [32]. There are two main routes which induce acetate formation from pyruvate in *E. coli*: (1) phosphotransacetylase (*pta*) and acetate kinase (*ackA*) catalyze acetyl-CoA to acetate; (2) pyruvate oxidase (*poxB*) catalyzes pyruvate to acetate [33]. In the previous study, Rhie et al. reported that deletion of *ackA* and *pta* exhibited substantially reduced levels of 3HV formation and thus would reduce the content of other odd-chain monomers. Hence, the *ackA* and *pta* genes were also confirmed to be essential for odd-chain monomer production [34]. Additionally, the enzyme pyruvate oxidase (PoxB) is regarded as a major candidate responsible for catalyzing the decarboxylation of pyruvate to form acetate and CO_2_. Several approaches have been performed to reduce the competitive acetate flux through the PoxB pathways [33] and to increase the acetyl-CoA pool [35]. Therefore, the *poxB* gene was deleted to reduce acetate secretion and provide more acetyl-CoA precursors for mcl-PHA biosynthesis in *E. coli*. Previous studies have indicated that intracellular ATP concentration was 18% higher in a *pflB* mutant growing under aerobic conditions, compared to the parental strain [36], which may be beneficial for biomass production and protein expression. According to this, gene deletion of *pflB* encoding pyruvate formate-lyase was also performed. After the double knockout, we constructed the engineered strain LZ08, and then the plasmids pQQ05 and pZQ06 were introduced into LZ08. As a consequence, when cultivation of the strain with the supplement of 30 g/L glucose and 2 g/L propionate, higher content of the mcl-PHA polymers was detected at 6.23 wt% with the molar content of odd-numbered monomers accounting for 20.03 mol% in batch cultivation, which means 456.04 mg/L mcl-PHA in the culture medium. The dominant one in even-chain monomers is also 3HO and 3HHp fraction accounts for the highest amount in odd-numbered monomers (Fig. 6a, b). Compared to other strategies such as fatty acid β-oxidation and de novo biosynthesis, the yield of mcl-PHA containing odd-numbered monomers was the highest and the category of monomer composition accumulated in a single strain was more various. These characteristics of monomer composition distribution may endow the mcl-PHA polyesters with novel mechanical properties and broader applications. Although further deletion of *poxB* and *pflB* in the engineered strain enhanced the odd-numbered monomer production, the even-numbered monomers still were the major constituents (Fig. 6a). It seemed that the Km for propionyl-CoA of thiolase (YqeF) was much higher than that of acetyl-CoA. As shown in Fig. 6b, we investigated the cell growth performance and glucose consumption of the strain LZ08 harboring pQQ05 and pZQ06. Cultivation of this engineered strain, we found that there was a short growth lag phase at the initial stage. After 10 h, the strain grew rapidly and reached its maximum cell dry weight of 7.32 g/L at 64 h (Fig. 6b). Meanwhile, we detected the acetate secretion in the medium in the shake flask experiments. As shown in Fig. 6c, acetate secretion dropped by 56% after the double deletion from 4.18 g/L to 1.84 g/L in the culture of the strain LZ08 (pQQ05, pZQ06) and the statistical analysis showed the marked decrease of acetate secretion compared with the strain LZ05 (pQQ05, pZQ06). This indicated that the double knockout was of great significance for cell growth and mcl-PHA accumulation in cells. Further studies will be needed to enhance content of the odd-numbered monomers in the mcl-PHA production and augment the biosynthesis of intracellular propionyl-CoA from renewable and structurally-unrelated carbon source directly.

## Conclusions

It has been a challenging task to synthesize mcl-PHA copolymers for a long time, especially for synthesizing even- and odd-chain mcl-PHA monomers equal to or longer than C8. There was no research reported that could make PHA copolyesters consisting of C6–C14 even- and odd-chain monomers. However, the fatty acid β-oxidation reversal was successfully utilized to generate the intermediates of mcl-PHA from renewable feedstocks in this study. By integrating two parallel precursor-supplying modules, the *E. coli* strain was confirmed to produce mcl-PHA containing both odd- and even-chain monomers efficiently. After optimization of the odd-numbered monomer module and the chassis, *E. coli* was found to synthesize mcl-PHA up to 6.23 wt% harboring odd-numbered monomers about 20.03 mol% from glucose and propionate. To the best of our knowledge, this is by far the first report on the novel mcl-PHA production both with even- and odd-numbered monomers with the highest yield. When grown on glucose and other related fatty acids, the recombinant *E. coli* was capable of producing other molar ratios of the monomers. This allows for generation of more and more PHA smart materials with diverse properties. Therefore, the engineered *E. coli* will be recruited as potential valuable and intelligent cell factories for industrial production to meet various applications.

## Methods

### Microbial strains and media

Table 1 lists the various strains and plasmids used in this study. The *E. coli* LS5218 strain [*fadR*, *atoC*(Con)], which constitutively expresses the enzymes of fatty acid β-oxidation pathway, allows expression of various pathway genes cloned into pTrc99a and pBBR1MCS2 vectors upon induction with isopropyl-β-d-1-thiogalactopyranoside (IPTG) [38]. The IPTG was added when the cells had reached an optical density at a wavelength of 600 nm (OD_600_) of 0.6. The PHA-producing strain LZ05 with deletion of the genes *ptsG*, *tesB* and *yciA* was described previously [23]. The *E. coli* DH5α strain served as the host strain for subsequent construction and propagation of various PHA-producing plasmids. During the recombinant plasmid construction, strains were cultivated in Luria–Bertani (LB) medium (10 g/L tryptone, 5 g/L yeast extract, 10 g/L NaCl). For gene knockout, SOB medium (20 g/L tryptone, 5 g/L yeast extract, 0.5 g/L NaCl, 10 mM MgCl_2_ and 2.5 mM KCl) was utilized.

**Table 1:** Strains and plasmids used in this study

Strains and plasmids	Relevant genotype/property	Source or references
Strains		
*E. coli* DH5a	F^−^, *endA1*, *hsdR17*, (*rk*^−^, *mk*^+^), *supE44*, *thi*-*1*, λ^−^, *recA1*, *gyrA96*, Δ*lacU169* (Φ80 *lacZ* Δ*M15*)	Laboratory stock
*E. coli* LS5218	F^+^, *fad*R601, *ato*C512 (Const)	Laboratory stock
LZ05	*E. coli* LS5218 Δ*ptsG*::FRT Δ*tesB*::FRT Δ*yciA*::FRT	Zhuang et al. [23]
LZ08	*E. coli* LS5218 Δ*ptsG*::FRT Δ*tesA*::FRT Δ*pflB*::FRT Δ*poxB*::FRT	This study
Plasmids		
pBBR1MCS-2	*lacPOZ mobRP4*, low-copy-no. cloning vector; Km^R^	Kovach et al. [37]
pQQ05	pTrc99a derivative, *yqeF* and *fadB* from *E. coli* MG1655, *phaJ1* _*Pa*_ and *phaC2* _*Pa*_ from *P. aeruginosa* PAO1, *ter* from *Treponema denticola*	Zhuang et al. [23]
pZQ01	pBBR1MCS2-prpP; pBBR1MCS-2 derivative, *prpP* from *R. eutropha* H16	This study
pZQ02	pBBR1MCS2-acs; pBBR1MCS-2 derivative, *acs* from *E. coli* MG1655	This study
pZQ03	pBBR1MCS2-prpE; pBBR1MCS2-derivative, *prpE* from *R. eutropha* H16	This study
pZQ04	pBBR1MCS2-pct; pBBR1MCS-2 derivative, *pct* from *R. eutropha* H16	This study
pZQ05	pBBR1MCS2-prpP-acs; pBBR1MCS-2 derivative, *prpP* from *R. eutropha* H16 and *acs* from *E. coli* MG1655	This study
pZQ06	pBBR1MCS2-prpP-prpE; pBBR1MCS2-derivative, *prpP* and *prpE* from *R. eutropha* H16	This study
pZQ07	pBBR1MCS2-prpP-pct; pBBR1MCS2-derivative, *prpP* and *pct* from *R. eutropha* H16	This study

### Plasmid construction

For the even-chain monomer supply, the construction of plasmid pQQ05 has been previously described [23]. Briefly, the genes *yqeF*, *fadB*, *phaJ1*_*Pa*_, *ter* and *phaC2*_*Pa*_ were all cloned and ligated into the corresponding sites of pTrc99a which were cut with the same restriction enzymes stepwise to generate plasmid pQQ05.

The construction of odd-chain monomer generation pathway was as follows. The codon-optimized *prpP* gene was cloned into the pBBR1MCS2 vector between the *Kpn*I and *Bam*HI sites to construct the plasmid of pZQ01. Later, in order to form the plasmid pBBR1MCS2-acs, namely pZQ02, the *acs* gene amplified via polymerase chain reaction (PCR) using *E. coli* MG1655 genomic DNA (gDNA) as template was also inserted into the pBBR1MCS2. The *prpE* and *pct* fragments amplified from *R. eutropha* H16 gDNA with primers prpE-F/prpE-R and pct-F/pct-R were separately ligated into the pBBR1MCS2 to yield the plasmids pZQ03 and pZQ04. Subsequently, co-expression of two genes *prpP* and *acs*, *prpP* and *prpE*, *prpP* and *pct* in the pBBR1MCS2 was utilized to form the plasmids pZQ05, pZQ06 and pZQ07, respectively. All of the genes were under the control of the lac promoter with separated ribosomal binding site located upstream of each gene to facilitate the translation. The *R. eutropha* H16 template used for these PCR reactions was isolated using the TIANamp Bacterial DNA Kit (TIANGEN BIOTECH, China). The primers used to amplify different fragments for cloning reactions are listed in Additional file 1: Table S1.

In all cases, PCR was performed using an S1000 Thermal Cycler (Bio-Rad, USA). PrimeSTAR HS DNA polymerase was purchased from Takara (Tokyo, Japan), restriction endonucleases were from Fermentas/Thermo Scientific (Pittsburgh, USA), and T4 DNA ligase was from New England Biolabs (Ipswich, USA). Propagated plasmids were prepared by TIANGEN Plasmid Mini Extraction Kit (TIANGEN BIOTECH, China), and restriction enzyme-digested products were purified using an E.Z.N.A.™ Gel Extraction Kit (Omega, USA). DNA sequencing of all constructed plasmids were performed by Liuhe BGI Tech Co. Ltd (Beijing, China). All of the constructed plasmids were transformed into the strain LZ05 and the optimum double plasmids were then transformed into the strain LZ08 according to standard procedures [39].

### Gene knockout

The gene *pflB* which encodes pyruvate formate lyase was knocked out by the one-step inactivation method as described previously [40] and *poxB* encoding pyruvate oxidase was knocked out by linearized DNA fragments with extending homologous sequence [41]. First, the linerized DNA fragments with the FLP recognition target sites and 39 bp homologous sequences were obtained via PCR using pKD4 (Km^R^) as a template and pflB-F/pflB-R as primers. After the DNA gel extraction, the purified PCR product was electroporated into the host cells which carried the plasmid pKD46, and then *E. coli* LZ05 was induced by 0.3% (w/v) l-arabinose to express the λ Red system. The positive transformants were selected and identified by colony PCR using the primers pflB-test-F/pflB-test-R. Regarding the *poxB* deletion, primers poxB-F/poxB-R and chromosomal DNA of the strain QZ1111 were applied to amplify the linearized DNA fragments for *poxB*. The deletion procedure of *poxB* gene was as follows. After DpnI digestion, the PCR products were then purified and electroporated into the competent strain *E. coli* LZ05 containing the plasmid pKD46. Transformant cells were selected in solid LB medium (1% tryptone, 0.5% yeast extract, 1% NaCl, and 1.5% agar powder) containing chloramphenicol (Cm^R^). Candidate clones were screened by PCR employing primers poxB-F/poxB-R. The PCR products were ultimately sequenced in Liuhe BGI Tech Co. Ltd (Beijing, China) if necessary. After removing pKD46, the corresponding Km^R^ or Cm^R^ cassette was removed with the helper plasmid pCP20. The plasmids pKD46 and pCP20 were eliminated by overnight cultivation at 42 °C. Finally, the strain LZ08 with the above two gene inactivation was generated.

### Cultivation condition

The medium of shake flask study contains 10 g/L tryptone, 5 g/L yeast extract, 30 mM NH_4_Cl, 5 mM (NH_4_)_2_SO_4_, 1.48 mM Na_2_HPO_4_, and 100 μM FeSO_4_ supplemented with 125 mM MOPS.

For all shake flask experiments, single colony was inoculated into 5 mL LB broth and grown at 37 °C overnight. 0.5 mL pre-culture was inoculated to 300 mL Erlenmeyer flask containing 50 mL LB and cultivated for 8 to 10 h and then 1% (v/v) seed inoculum for shake flask cultivation was incubated in 50 mL fermentation medium. When all liquid fermentation medium (50 mL) was incubated in 300 mL conical flasks at 37 °C with an agitation of 250 rpm to an optical density at 600 nm (OD_600_) of 0.6–0.8, 1 mM IPTG was added to the culture broth as an inducer. After induction, 30 g/L glucose was supplied as the sole carbon source at the appropriate time and then fermented for 72 h at 30 °C with shaking at 250 rpm. When necessary, ampicillin (100 μg/mL), kanamycin (50 μg/mL) or chloramphenicol (25 μg/mL) was added to the medium to maintain the stability of the plasmids. After cultivation, cells were gathered by centrifugation at 12,000 rpm for 15 min, washed with water twice and treated with ethanol once and then lyophilized.

### PHA production analysis

The content and monomer compositions of intracellular accumulated PHA were analyzed by gas chromatography (GC) as described previously [42]. PHA content was defined as the percent ratio of PHA concentration to CDW. Liquid culture was centrifuged to obtain the supernatant and cellular biomass. 15 mg lyophilized cells were subjected to methanolysis in the presence of 1 mL of chloroform and 1 mL of 3% (v/v) sulfuric acid in methanol for 1 h at 100 °C. The samples were cooled to room temperature and then 1 mL of distilled water was added in order to extract the cell debris that is soluble in the aqueous phase. 10 mg/mL pentadecanoic acid in ethanol was added as an internal standard. The mixture was vortexed and centrifuged at 12,000 rpm for 10 min. After the layer separation, the organic (chloroform) phase (500 μL) was transferred to another new vial and analyzed using a Shimadzu GC2010 gas chromatograph (Kyoto, Japan) equipped with an AOC-20i auto-injector and a RestekRxi^®^-5 column. PHA standard samples were dissolved in chloroform and also analyzed according to the method above by GC. The temperature program used was as follows: 80 °C hold for 1 min, ramp from 60 to 230 °C at 10 °C per min and a final hold at 230 °C for 10 min [23].

### Cell growth, glucose consumption and acetate assimilation analyses

Cell growth was monitored by measuring OD_600_ utilizing a spectrophotometer (Shimazu, Japan). Glucose and acetate were quantitatively analyzed by high-performance liquid chromatography (HPLC) (Shimazu, Japan) which equipped with a refractive index detector (RID-10A) and an Ion Exclusion column (Bio-Rad, HPX-87H). The samples were first centrifuged at 12,000 rpm for 10 min, and then the supernatant was filtrated with a 0.22 μm filter membrane. 5 mM sulfuric acid was utilized as the mobile phase of HPLC with the flow rate of 0.6 mL/min and the utilized column temperature was 65 °C.

### Statistical analyses

All data examined were expressed as mean ± SD. Statistical analyses of the data were carried out using two-tailed Student’s t-test between two groups, and one-way ANOVA followed by the post hoc Tukey’s test for multiple groups. *P* < 0.05 was considered significant. The * denotes *P* < 0.05, the *** denotes *P* < 0.001.

## Additional file

**Additional file 1: Table S1.** Oligonucleotides used in this study.

## Data Availability

All data generated or analyzed during this study are included in this published article.

## Abbreviations

PEP: phosphoenolpyruvate
Glucose-6-P: glucose-6-phosphate
2[H]: NADH/NADPH/ferredoxin
Ext: extracellular
Int: intracellular
CDW: cell dry weight
3HHx: 3-hydroxyhexanoate
3HHp: 3-hydroxyheptanoate
3HO: 3-hydroxyoctanoate
3HN: 3-hydroxynonanoate
3HD: 3-hydroxydecanoate
3HUD: 3-hydroxyundecanoate
3HDD: 3-hydroxydodecanoate
3HTRD: 3-hydroxytridecanoate
3HTD: 3-hydroxytetradecanoate

## References

[1] Chen GQ, Jiang XR (2018). Engineering microorganisms for improving polyhydroxyalkanoate biosynthesis. Curr Opin Biotechnol.

[2] Zhuang QQ (2017). Progress in synthetic biology of *Escherichia coli* to produce polyhydroxyalkanoates. Chinese J Bioproc Eng..

[3] Patel SKS, Sandeep K, Singh M, Singh GP, Lee JK, Bhatia SK, Kalia VC, Kalia VC (2019). Biotechnological application of polyhydroxyalkanoates and their composites as anti-microbials agents. Biotechnological applications of polyhydroxyalkanoates.

[4] Wang Q, Zhuang QQ, Liang QF, Qi QS (2013). Polyhydroxyalkanoic acids from structurally-unrelated carbon sources in *Escherichia coli*. Appl Microbiol Biotechnol.

[5] Wang Y, Chung A, Chen GQ (2017). Synthesis of medium-chain-length polyhydroxyalkanoate homopolymers, random copolymers, and block copolymers by an engineered strain of *Pseudomonas entomophila*. Adv Healthc Mater..

[6] Chen GQ, Hajnal I, Wu H, Lv L, Ye JW (2015). Engineering biosynthesis mechanisms for diversifying polyhydroxyalkanoates. Trends Biotechnol.

[7] Singh AK, Srivastava JK, Chandel AK, Sharma L, Mallick N, Singh SP (2019). Biomedical applications of microbially engineered polyhydroxyalkanoates: an insight into recent advances, bottlenecks and solutions. Appl Microbiol Biotechnol.

[8] Wang HH, Li XT, Chen GQ (2009). Production and characterization of homopolymer polyhydroxyheptanoate (P3HHp) by a *fadBA* knockout mutant *Pseudomonas putida* KTOY06 derived from *P. putida* KT2442. Process Biochem.

[9] Qi QS, Rehm BH, Steinbüchel A (1997). Synthesis of poly(3-hydroxyalkanoates) in *Escherichia coli* expressing the PHA synthase gene *phaC2* from *Pseudomonas aeruginosa*: comparison of PhaC1 and PhaC2. FEMS Microbiol Lett.

[10] Razaif-Mazinah MRM, Anis SNS, Harun HI, Rashid KA, Annuar MSM (2017). Unusual poly(3-hydroxyalkanoate) (PHA) biosynthesis behavior of *Pseudomonas putida* Bet001 and *Delftia tsuruhatensis* Bet002 isolated from palm oil mill effluent. Biotechnol Appl Biochem.

[11] Gillis J, Ko K, Ramsay JA, Ramsay BA (2017). Potential for mcl-PHA production from nonanoic and azelaic acids. Can J Microbiol.

[12] Tseng HC, Prather KLJ (2012). Controlled biosynthesis of odd-chain fuels and chemicals via engineered modular metabolic pathways. Proc Nat Acad Sci..

[13] Ma YC, Cui Y, Du LH, Liu XQ, Xie XX, Chen N (2018). Identifcation and application of a growth-regulated promoter for improving l-valine production in *Corynebacterium glutamicum*. Microb Cell Fact.

[14] Ma WL, Liu YF, Lv XQ, Li JH, Du GC, Liu L (2019). Combinatorial pathway enzyme engineering and host engineering overcomes pyruvate overflow and enhances overproduction of N-acetylglucosamine in *Bacillus subtilis*. Microb Cell Fact.

[15] Huccetogullari D, Luo ZW, Lee SY (2019). Metabolic engineering of microorganisms for production of aromatic compounds. Microb Cell Fact.

[16] Cai P, Gao JQ, Zhou YJ (2019). CRISPR-mediated genome editing in non-conventional yeasts for biotechnological applications. Microb Cell Fact.

[17] Agnew DE, Stevermer AK, Youngquist JT, Pfleger BF (2012). Engineering *Escherichia coli* for production of C12-C14 polyhydroxyalkanoate from glucose. Metab Eng.

[18] Wang Q, Tappel RC, Zhu C, Nomura CT (2012). Development of a new strategy for production of medium-chain-length polyhydroxyalkanoates by recombinant *Escherichia coli* via inexpensive non-fatty acid feedstocks. Appl Environ Microbiol.

[19] Li ZJ, Qiao KJ, Che XM, Stephanopoulos G (2017). Metabolic engineering of *Escherichia coli* for the synthesis of the quadripolymer poly(glycolate-co-lactate-co-3-hydroxybutyrate-co-4-hydroxybutyrate) from glucose. Metab Eng.

[20] Foong CP, Lakshmanan M, Abe H, Taylor TD, Foong SY, Sudesh K (2018). A novel and wide substrate specific polyhydroxyalkanoate (PHA) synthase from unculturable bacteria found in mangrove soil. J Polym Res.

[21] Clomburg JM, Contreras SC, Chou A, Siegel JB, Gonzalez R (2018). Combination of type II fatty acid biosynthesis enzymes and thiolases supports a functional β-oxidation reversal. Metab Eng.

[22] Kallscheuer N, Polen T, Bott M, Marienhagen J (2017). Reversal of β-oxidative pathways for the microbial production of chemicals and polymer building blocks. Metab Eng.

[23] Zhuang QQ, Wang Q, Liang QF, Qi QS (2014). Synthesis of polyhydroxyalkanoates from glucose that contain medium-chain-length monomers via the reversed fatty acid β-oxidation cycle in *Escherichia coli*. Metab Eng.

[24] Horng YT, Chien CC, Huang CT, Wei YH, Chen SY, Lan JCW, Soo PC (2013). Biosynthesis of poly(3-hydroxybutyrate-co-3-hydroxyvalerate) with co-expressed propionate permease (*prpP*), beta-ketothiolase B (*bktB*), and propionate-CoA synthase (*prpE*) in *Escherichia coli*. Biochem Eng J.

[25] Yang JE, Choi YJ, Lee SJ, Kang KH, Lee H, Oh YH, Lee SH, Park SJ, Lee SY (2014). Metabolic engineering of *Escherichia coli* for biosynthesis of poly(3-hydroxybutyrate-co-3-hydroxyvalerate) from glucose. Appl Microbiol Biotechnol.

[26] Volodina E, Schürmann M, Lindenkamp N, Steinbüchel A (2013). Characterization of propionate CoA-transferase from *Ralstonia eutropha* H16. Appl Microbiol Biotechnol.

[27] Liu M, Ding YM, Chen HL, Zhao Z, Liu HZ, Xian M, Zhao G (2017). Improving the production of acetyl-CoA-derived chemicals in *Escherichia coli* BL21 (DE3) through *iclR* and *arcA* deletion. BMC Microbiol.

[28] Liu FY, Gu J, Wang XD, Zhang XE, Deng JY (2014). Acs is essential for propionate utilization in *Escherichia coli*. Biochem Biophys Res Commun.

[29] Lindenkamp N, Schürmann M, Steinbüchel A (2013). A propionate CoA-transferase of *Ralstonia eutropha* H16 with broad substrate specificity catalyzing the CoA thioester formation of various carboxylic acids. Appl Microbiol Biotechnol.

[30] Wong MS, Causey TB, Mantzaris N, Bennett GN, San KY (2008). Engineering poly(3-hydroxybutyrate-co-3-hydroxyvalerate) copolymer composition in *E. coli*. Biotechnol Bioeng.

[31] Peng L, Shimizu K (2003). Global metabolic regulation analysis for *Escherichia coli* K12 based on protein expression by 2-dimensional electrophoresis and enzyme activity measurement. Appl Microbiol Biotechnol.

[32] Sandoval NR, Mills TY, Zhang M, Gill RT (2010). Elucidating acetate tolerance in *E. coli* using a genome-wide approach. Metab Eng.

[33] Dittrich CR, Vadali RV, Bennett GN, San KY (2005). Redistribution of metabolic fluxes in the central aerobic metabolic pathway of* E. coli *mutant strains with deletion of the *ackA*-*pta* and *poxB* pathways for the synthesis of isoamyl acetate. Biotechnol Progr.

[34] Rhie HG, Dennis D (1995). The function of *ackA* and *pta* genes is necessary for poly(3-hydroxybutyrate-co-3-hydroxyvalerate) synthesis in recombinant* pha*+ *Escherichia coli*. Can J Microbiol.

[35] Vadali RV, Horton CE, Rudolph FB, Bennett GN, San KY (2004). Production of isoamyl acetate in *ackA*-*pta* and/or* ldh* mutants of* Escherichia coli *with overexpression of yeast ATF2. Appl Microbiol Biotechnol.

[36] Zhu J, Shimizu K (2004). The effect of *pfl *gene knockout on the metabolism for optically pure D-lactate production by* Escherichia coli*. Appl Microbiol Biotechnol.

[37] Kovach ME, Elzer PH, Hill DS, Robertson GT, Farris MA, Roop II RM, Peterson KM (1995). Four new derivatives of the broad-host-range cloning vector pBBR1MCS, carrying different antibiotic-resistance cassettes. Gene.

[38] Spratt SK, Ginsburgh CL, Nunn WD (1981). Isolation and genetic characterization of *Escherichia coli *mutants defective in propionate metabolism. J Bacteriol.

[39] Sambrook J, Russell DW (2001). Molecular cloning: a laboratory manual.

[40] Datsenko KA, Wanner BL (2000). One-step inactivation of chromosomal genes in *Escherichia coli* K-12 using PCR products. Proc Nat Acad Sci..

[41] Li MJ, Gu PF, Kang JH, Wang Y, Wang Q, Qi QS (2012). Extending homologous sequence based on the single gene mutants by one-step PCR for efficient multiple gene knockouts. Folia Microbiol.

[42] Kato M, Bao H, Kang CK, Fukui T, Doi Y (1996). Production of a novel copolyester of 3-hydroxybutyric acid and medium-chain-length 3-hydroxyalkanoic acids by *Pseudomonas* sp. 61-3 from sugars. Appl Microbiol Biotechnol.

