# Supplementary material for: Engineering the pathway in Escherichia coli for the synthesis of medium-chain-length polyhydroxyalkanoates consisting of both even- and odd-chain monomers
Source: Microb Cell Fact. 2019 Aug 13;18:135. doi: 10.1186/s12934-019-1186-x (PMC6693092; doi:10.1186/s12934-019-1186-x)
Supplement: Supplementary file 1 — Additional file 1: Table S1. Oligonucleotides used in this study. [file 12934_2019_1186_MOESM1_ESM.doc]

**Table S1.** Oligonucleotides used in this study

| Primers | Primer sequences (5’-3’) and restriction enzymes used |
| --- | --- |
| Plasmid construction | |
| prpP-F | CGGGGTACCCATGTCCTTTCTGATTGTTCT |
| prpP-R | CGCGGATCCTCACACCAGACCCGTCAGGAAAT |
| acs-F  acs-R  prpE-F | CGGGGTACCCATGAGCCAAATTCACAAACA  CGCGGATCCTTACGATGGCATCGCGATAG  CGGGGTACCCatgacggcaagccatgccgtgcaT |
| prpE-R | CGCGGATCCCTAGCCTTTCAGCGCTGCCTG |
| pct-F | CGGGGTACCCATGAAGGTGATCACCGCA |
| pct-R | CGCGGATCCTTACAGGTGCAGGGGCCCGGCCT |
| acs-F2 | CGCGGATCCAGGAGGTATAATTAATGAGCCAAATTCACAAACA |
| acs-R2 | GCTCTAGATTACGATGGCATCGCGATAG |
| prpE-F2 | CGCGGATCCAGGAGGTATAATTAatgacggcaagccatgccgtgcaT |
| prpE-R2 | GCTCTAGACTAGCCTTTCAGCGCTGCCTG |
| pct-F2 | CGCGGATCCAGGAGGTATAATTAATGAAGGTGATCACCGCA |
| pct-R2 | GCTCTAGATTACAGGTGCAGGGGCCCGGCCT |
| Gene deletion | |
| pflB-F | GCAGTAAATAAAAAATCCACTTAAGAAGGTAGGTGTTACGTGTAGGCTGGAGCTGCTTC |
| pflB-R | ATTGTACGCTTTTTACTGTACGATTTCAGTCAAATCTAAATGGGAATTAGCCATGGTCC |
| pflB-test-F | AGCGGTTTTGAGCACAGTATCGC |
| pflB-test-R | AATACAGGCAGCGCATCAGGCAG |
| poxB-F | ACTGGAGGCGCTGGAAAGCAATA |
| poxB-R | GGCACGCTACCGCTGGATAAAGT |

The restriction endonuclease digestion sites were underlined.
